# Generalization and Dilution of Association Results from European GWAS in Populations of Non-European Ancestry: The PAGE Study

**DOI:** 10.1371/journal.pbio.1001661

**Published:** 2013-09-17

**Authors:** Christopher S. Carlson, Tara C. Matise, Kari E. North, Christopher A. Haiman, Megan D. Fesinmeyer, Steven Buyske, Fredrick R. Schumacher, Ulrike Peters, Nora Franceschini, Marylyn D. Ritchie, David J. Duggan, Kylee L. Spencer, Logan Dumitrescu, Charles B. Eaton, Fridtjof Thomas, Alicia Young, Cara Carty, Gerardo Heiss, Loic Le Marchand, Dana C. Crawford, Lucia A. Hindorff, Charles L. Kooperberg, for the PAGE Consortium

**Affiliations:** 1 Division of Public Health Sciences, Fred Hutchinson Cancer Research Center, Seattle, Washington, United States of America; 2 Department of Genetics, Rutgers University, Piscataway, New Jersey, United States of America; 3 Department of Epidemiology and Carolina Center for Genome Sciences, University of North Carolina, Chapel Hill, North Carolina, United States of America; 4 Department of Preventive Medicine, Keck School of Medicine, University of Southern California/Norris Comprehensive Cancer Center, Los Angeles, California, United States of America; 5 Center for Child Health, Behavior, and Development, Seattle Children's Research Institute, Seattle, Washington, United States of America; 6 Department of Statistics & Biostatistics, Rutgers University, Piscataway, New Jersey, United States of America; 7 Department of Biochemistry and Molecular Biology, The Pennsylvania State University, University Park, Pennsylvania, United States of America; 8 Translational Genomics Research Institute, Phoenix, Arizona, United States of America; 9 Department of Biology & Environmental Science at Heidelberg University, Tiffin, Ohio, United States of America; 10 Department of Molecular Physiology and Biophysics, Center for Human Genetics Research, Vanderbilt University, Nashville, Tennessee, United States of America; 11 Department of Family Medicine, Brown University, Pawtucket, Rhode Island, United States of America; 12 Division of Biostatistics & Epidemiology, Department of Preventive Medicine, College of Medicine, The University of Tennessee Healthy Science Center, Memphis, Tennessee, United States of America; 13 Epidemiology Program, University of Hawaii Cancer Center, Honolulu, Hawaii, United States of America; 14 Division of Genomic Medicine, National Human Genome Research Institute, National Institutes of Health, Bethesda, Maryland, United States of America; Georgia Institute of Technology, United States of America

## Abstract

The vast majority of genome-wide association study (GWAS) findings reported to date are from populations with European Ancestry (EA), and it is not yet clear how broadly the genetic associations described will generalize to populations of diverse ancestry. The Population Architecture Using Genomics and Epidemiology (PAGE) study is a consortium of multi-ancestry, population-based studies formed with the objective of refining our understanding of the genetic architecture of common traits emerging from GWAS. In the present analysis of five common diseases and traits, including body mass index, type 2 diabetes, and lipid levels, we compare direction and magnitude of effects for GWAS-identified variants in multiple non-EA populations against EA findings. We demonstrate that, in all populations analyzed, a significant majority of GWAS-identified variants have allelic associations in the same direction as in EA, with none showing a statistically significant effect in the opposite direction, after adjustment for multiple testing. However, 25% of tagSNPs identified in EA GWAS have significantly different effect sizes in at least one non-EA population, and these differential effects were most frequent in African Americans where all differential effects were diluted toward the null. We demonstrate that differential LD between tagSNPs and functional variants within populations contributes significantly to dilute effect sizes in this population. Although most variants identified from GWAS in EA populations generalize to all non-EA populations assessed, genetic models derived from GWAS findings in EA may generate spurious results in non-EA populations due to differential effect sizes. Regardless of the origin of the differential effects, caution should be exercised in applying any genetic risk prediction model based on tagSNPs outside of the ancestry group in which it was derived. Models based directly on functional variation may generalize more robustly, but the identification of functional variants remains challenging.

## Abstract 2

A multi-ethnic study demonstrates that the extrapolation of genetic disease risk models from European populations to other ethnicities is compromised more strongly by genetic structure than by environmental or global genetic background in differential genetic risk associations across ethnicities.

## Author Summary

The number of known associations between human diseases and common genetic variants has grown dramatically in the past decade, most being identified in large-scale genetic studies of people of Western European origin. But because the frequencies of genetic variants can differ substantially between continental populations, it's important to assess how well these associations can be extended to populations with different continental ancestry. Are the correlations between genetic variants, disease endpoints, and risk factors consistent enough for genetic risk models to be reliably applied across different ancestries? Here we describe a systematic analysis of disease outcome and risk-factor–associated variants (tagSNPs) identified in European populations, in which we test whether the effect size of a tagSNP is consistent across six populations with significant non-European ancestry. We demonstrate that although nearly all such tagSNPs have effects in the same direction across all ancestries (i.e., variants associated with higher risk in Europeans will also be associated with higher risk in other populations), roughly a quarter of the variants tested have significantly different magnitude of effect (usually lower) in at least one non-European population. We therefore advise caution in the use of tagSNP-based genetic disease risk models in populations that have a different genetic ancestry from the population in which original associations were first made. We then show that this differential strength of association can be attributed to population-dependent variations in the correlation between tagSNPs and the variant that actually determines risk—the so-called functional variant. Risk models based on functional variants are therefore likely to be more robust than tagSNP-based models.

## Introduction

In the past six years, genome-wide association studies (GWAS) have revealed thousands of common polymorphisms (tagSNPs) associated with a wide variety of traits and diseases, particularly as study sample sizes have increased from thousands to hundreds of thousands of subjects. Typically GWAS analyses stratify on genetic ancestry, because many polymorphism allele frequencies differ by ancestral group, easily producing false positive associations for traits that also correlate with genetic ancestry. The large majority of GWAS results reported to date derive from analyses in populations of European ancestry (EA) [1],[2]. Although GWAS in Asian populations in particular are becoming more common [3]–[6], it remains important to understand the degree to which the magnitude and direction of allelic effects generalize across diverse populations [7]–[10]. The multi-ethnic PAGE consortium [11] provides a unique opportunity to assess GWAS generalization across multiple non-EA populations and multiple traits.

## Results and Discussion

Subject and genotyping panel selection for the PAGE consortium have been described elsewhere [11],[12]. In brief, a panel of 68 common polymorphisms previously reported to associate with body mass index (BMI) [13], type 2 diabetes (T2D) [14], or lipid levels [15] was genotyped in up to 14,492 self-reported African Americans (AA), 8,202 Hispanic Americans (HA), 5,425 Asian Americans (AS), 6,186 Native Americans (NA), 1,801 Pacific Islanders (PI), and 37,061 EA (for details, see Materials and Methods, Table S1 and Table S2). We also analyzed a subset of 5863 AA from PAGE who were genotyped on the Illumina Metabochip, which contains approximately 200,000 SNPs densely focused on 257 regions with reported GWAS associations to traits that include lipids, BMI, and T2D [16].

For a replication analysis it would be overly conservative to use the Bonferroni correction, so the Benjamini-Hochberg method [17] was applied to assess replication of previous EA reports in the PAGE EA population. Reported effects in EA were replicated for 51 out of the 68 index SNPs at a 5% FDR. Power to replicate at most of these 68 SNPs far exceeded 80%; 16 of the 17 SNPs that did not replicate exceeded 80% power to replicate the reported effect size, and the 17th exceeded 70% power, as described previously [13]–[15]. The originally reported effect sizes tend to be less extreme for these seventeen index SNPs, but in 63 out of 79 comparisons between non-EA and EA populations involving these 17 SNPs, the direction of effect was the same in EA and non-EA groups (*p*<10^−5^ for the null hypothesis of random effects in either direction, data in Table 1 column “Index SNPs Not Replicated in EA”). Only 79 of the 85 possible pairwise comparisons against EA were assessed, because some of the 17 SNPs were not genotyped in all five non-EA populations. Thus, it appears likely that most of the 17 failures to replicate represent weak effects that were underpowered in PAGE EA, rather than false-positive primary reports. Therefore, all 68 index SNPs were carried forward in the generalization analysis.

**Table 1:** Summary of direction and strength of β relative to EA.

			Direction relative to EA^a^		
		All Index SNPs	Index SNPs Replicated in EA	Index SNPs Not Replicated in EA	Strength Relative to EA
Pop.	N^b^	Same∶Opposite^c^	Same∶Opposite^c^	Same∶Opposite^c^	Stronger∶Weaker^d^
AA	14,492	57∶11***	43∶8***	14∶3	0∶12**
HA	8,202	60∶8***	46∶5***	14∶3	0∶0
AS	5,425	45∶21**	34∶15*	11∶6	0∶0
NA	6,186	45∶10***	35∶8***	10∶2	0∶2
PI	1,801	48∶14***	34∶12***	14∶2	1∶0

a *p* values were computed from the binomial sign test against null expectation of 50% in same direction, not adjusted for multiple tests.

b Maximum number of samples per population. Not all SNPs were genotyped in all PAGE substudies; detailed numbers genotyped per variant are available in Table S2.

c Although some effects were observed where the sign of the coefficient differed between EA and AA, in none of these cases were both coefficients significantly different from zero, so no significantly opposite effects were observed in any non-EA population.

d Strength was evaluated only for index SNPs that replicated in EA, *and* showed differential effects in the non-EA population (ß_pop_≠ß_EA_). *p* values computed from the binomial sign test against null expectation of 50% stronger, not adjusted for multiple tests.

* *p*<0.05,

** *p*<0.01,

*** *p*<0.001.

**Figure 1:**
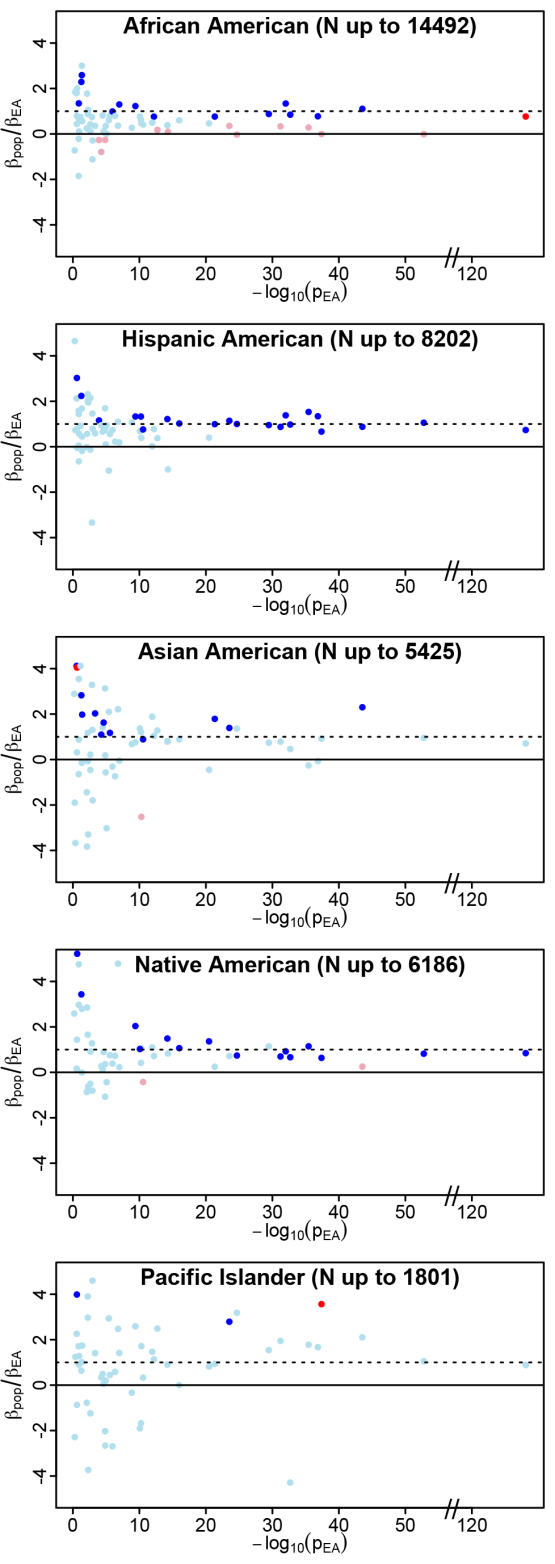
Generalization analysis in the PAGE populations. We plot the ratio of 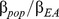 on the *y*-axis as an indicator of both consistency of direction (positive values are consistent with effects in the same direction) and relative magnitude of effect (consistent but weaker effects in the non-EA will have ratios between 0 and 1). The *p* value for trait association in the PAGE European American population (p_EA_) is an indicator of the strength of the original association. For each index SNP, we plot 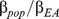 against −log_10_(p_EA_). Data points are colored as follows: ambiguous SNPs are light blue (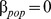 and 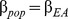), strictly generalized SNPs are dark blue (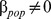 and 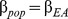), differentially generalized SNPs are dark red (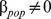 and 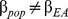), and differential SNPs are pink (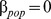 and 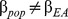). The *y*-axis has been constrained to (−4,4) for illustrative purposes; some loci yielded 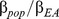 ratios outside this range, but p_EA_>0.05 for all of these. As expected, larger non-EA populations show less scatter in 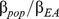 than the smaller non-EA populations (particularly Pacific Islanders), consistent with more precise estimates of 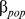 in the larger non-EA populations. Two clear trends are apparent in these plots: first, a trend toward 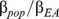 ratios greater than zero in all populations, especially for stronger effects in EA (−log10(p_EA_)>10), reflecting consistency of direction between EA and non-EA populations. Second, a trend toward ratios greater than zero but less than one is observed in African Americans, representing the trend toward dilution in this population, relative to EA. The second trend is not apparent in the other non-EA populations. Similar plots of 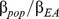 against observed allele frequency in the non-EA populations demonstrate that the allele frequency distribution for differential observations in AA is not different from the distribution of either ambiguous or strictly generalized loci, so the significantly diluted effects are not attributable to variants with low allele frequency in this population (Figure S1).

In all non-EA groups, we observe significantly more effects in the same direction as in EA than expected under the null hypothesis, ranging from 68% in Asians to 88% in Hispanics (*p*<0.001 in all non-EA groups, Table 1 and Figure 1). Even in the relatively small Pacific Islander population (*N* = 1801), where only four index SNPs were significantly associated with reported traits, 48 out of 62 effects were in the same direction as EA (*p*<0.001), so in larger samples from this population we would expect additional loci to generalize. Although a higher proportion of effects in the opposite direction of EA was observed in Asians and Pacific Islanders, the opposite effects were neither significantly different from no effect, nor significantly different from the observed effect in the EA population. This suggests that the greater number of effects in the opposite direction observed in these smallest groups simply reflects greater uncertainty in estimating effect sizes for these populations, rather than any true trend toward opposite effects. The proportion of effects in the same direction as EA was similar across all non-EA populations, suggesting that for at least 70% of index SNPs, a significant effect in a consistent direction will ultimately be observed in non-EA populations of adequate size.

Whereas the direction of effect was consistent between EA and non-EA populations, the magnitude of effect varied considerably, consistent with prior meta-analyses of generalization [18]. Because effect sizes were correlated among non-EA populations, we applied the Benjamini-Hochberg method within each population to identify index SNPs with significantly inconsistent effects between EA and non-EA populations. Inconsistent effects (*β_pop_*≠*β_EA_* at 5% FDR) were observed for 17 of 68 index SNPs in at least one non-EA population (Table 2 and Table S2, see Box 1 for definitions). Inconsistent effects were most frequent in the AA population (12 out of 68 loci), but examples were also observed in Pacific Islanders and Native Americans. Although most effects were consistent between EA and non-EA populations, the relatively high frequency with which differential effects were observed in non-EA populations suggests that genetic risk models derived from GWAS in EA will predict risk less reliably in non-EA populations, particularly AA. Consequently, caution should be exercised in applying risk models based upon risk variants genotyped outside of the ethnic background in which they were derived [19], regardless of the factors causing the observed variation between populations,.

**Table 2:** Summary of generalization results.

Significance: ß_pop_ versus Null	Not Significant (ß_pop_ = 0)		Significant (ß_pop_≠0)		
Consistency: ß_pop_ versus ß_EA_	Consistent (ß_pop_ = ß_EA_)	Inconsistent (ß_pop_≠ß_EA_)		Consistent (ß_pop_ = ß_EA_)	
Population	Ambiguous^a^	Differential^a^	Differentially Generalized^a^	Strictly Generalized^a^	Total^b^
AA	42	11	1	14	68
HA	46	0	0	22	68
AS	52	1	1	12	66
NA	35	2	2^c^	16	55
PI	59	0	1^c^	2	62

a See Box 1 for definitions.

b Totals differ between populations because not all SNPs were genotyped in all populations.

c Not visible in Figure 1 because of very small ß_EA_.

**Box 1. Definitions**

**ß_pop_**: The effect size of a given SNP in linear or logistic regression models for a specific PAGE population. Where available and when allowed by the informed consent protocols, effect sizes were estimated in models that included estimated genetic ancestry, as previously reported (see Text S1).

**ß_EA_**: The effect size of a given SNP in the PAGE EA population. We use the PAGE EA effect size for comparisons to PAGE non-EA populations rather than the original report for two reasons: to minimize the impact of winner's curse on these comparisons, and because several of the SNPs genotyped in PAGE were proxies strongly correlated with the original tagSNP, and might not match the reported effect size.

We define **replicated** SNPs as SNPs with direction of effect consistent with the original report in EA, and significant ß_EA_ in PAGE (using α = 0.05 as the threshold for hypothesis rejection, unadjusted for multiple testing, as these are considered specific prior hypothesis being validated).

When comparing two populations, the **direction** of effect can be either the **same** (ß_pop_ and ß_EA_ are either both positive, or both negative) or **opposite** (either ß_pop_ or ß_EA_ is positive, and the other is negative). **Magnitude** of effect was evaluated only for SNPs that replicated in EA and can be either **stronger** (|ß_EA_|<|ß_pop_|), the **same** (|ß_EA_| = |ß_pop_|), or **weaker** (|ß_EA_|>|ß_pop_|).

In order to describe the generalization of EA findings to non-EA populations, SNPs are categorized in terms of (a) significance in the non-EA population and (b) consistency between non-EA and EA populations. Here we use the Benjamini-Hochberg procedure to adjust for testing up to 68 SNPs in each non-EA population.

**Significant** SNPs reject the null hypothesis of no effect in the non-EA population (**ß_pop_≠0**) at q = 0.05.

**Inconsistent** SNPs reject the hypothesis of equal effect size in EA and non-EA populations (**ß_pop_≠ß_EA_**) at q = 0.05.

Combining these parameters yields four categories of generalization:

- **Ambiguous** SNPs are neither significant in non-EA, nor inconsistent between non-EA and EA.
- **Differential** SNPs are not significant in non-EA, but are inconsistent between non-EA and EA.
- **Differentially Generalized** SNPs are significant in non-EA, and inconsistent between non-EA and EA.
- **Strictly Generalized** SNPs are significant in non-EA, and consistent between non-EA and EA.

Four index SNPs showed differentially generalized effects (ß_pop_≠ß_EA_ and ß_pop_≠0). Two of these did not replicate in EA (rs7578597 and rs7961581 for T2D in NA) so consistency of direction cannot accurately be inferred. Direction of effect in EA and non-EA was the same for the remaining two index SNPs; rs3764261 was significantly weaker for HDL in AA, and rs28927680 was significantly stronger for TG in Pacific Islanders. There were no observations of opposite effects where both the EA effect and the non-EA effect were significant.

Considering only the 15 SNPs with a significantly inconsistent effect between EA and at least one non-EA population, 14 of 15 diluted toward the null (*p*<0.01, Table 2), a trend driven by the AA population, where all 12 out of 12 significant inconsistencies were diluted. Expanding analysis to all 51 loci replicated in EA, regardless of whether a significant difference was observed between EA and non-EA at a given SNP, we observed a significant excess of effects diluted toward the null (ß_pop_/ß_EA_<1) in AA, HA, and NA populations (Table S5). Comparisons between non-EA populations revealed that diluted effect sizes were significantly more likely in AA than in any other non-EA population.

Given that differential effect sizes were observed for many tagSNPs, we sought to leverage the data in order to assess the relative contributions of several factors that might contribute to the significant trend toward diluted effects, including gene–environment interaction with an exposure that varies across populations (differential environment), differences in the correlation between the index SNP and the functional variant across populations (differential tagging), modulation of the index SNP effect by additional, population-specific polymorphism (differential genetic background), population-specific synthetic alleles (combinations of rare, functional alleles tagged by a single common tagSNP [20]), or some combination of these factors. It seems unlikely that differential environments would be much more frequent in AA than other non-EA populations, or that differential environment would consistently bias toward the null within AA. Differential tagging is consistent with differentially diluted effects in AA; because linkage disequilibrium extends over significantly shorter distances in African populations than in non-African populations [21],[22], common functional variants (or synthetic alleles) are likely to be less strongly tagged by the index tagSNPs in AA. Differential genetic background effects in AA would also be consistent with the high nucleotide diversity known to exist in this population. The rare functional variants contributing to synthetic alleles will tend to be younger than common variants, and therefore are more likely to be population-specific, so synthetic alleles are compatible with the trend toward dilution. Thus, although differential environmental effects cannot be excluded, the observed data are more consistent with differential tagging and/or differential genetic background effects, and synthetic alleles cannot be excluded.

Genetic background effects can be subdivided into modifying effects, where variants elsewhere in the genome directly alter the effect associated with a given index SNP, and interference effects, where secondary variants change the proportion of variance explained by the index SNP. Interfering functional variants with effects in the same direction as the index SNP would tend to dilute the apparent effect size at the index variant. The most likely source of such variants is the region surrounding an index SNP, as demonstrably functional variants already exist in that region. Although examples have been described of genes carrying both risk and protective mutations [23]–[25], others clearly exhibit trends toward risk alleles with similar effects (e.g., preferentially toward breast cancer risk alleles at *BRCA1* [26]). If the direction of effect for functional variants in a given region is consistently biased, then an increase in the number of interfering variants within a given population would be consistent with a trend toward dilution of index effects. The higher nucleotide diversity observed in African populations relative to non-African populations [27],[28] would be consistent with a greater burden of secondary functional variants in AA than other populations.

In order to assess contribution of the factors outlined above to differential effect sizes between EA and AA in the index tagSNP associations, high density genotype data were collected from a subset of the PAGE African American sample. The number of AA individuals used for index tagSNP analyses varied by phenotype, with an average of 7501 (Table S3). Similar data on other populations are currently unavailable, so only loci showing differential effects between EA and AA could be analyzed. Genotype data were collected using the Metabochip, a high density genotyping array commercially available from Illumina. Detailed methods for the Metabochip genotype data collection, calling, and quality control are available elsewhere [12].

**Figure 2:**
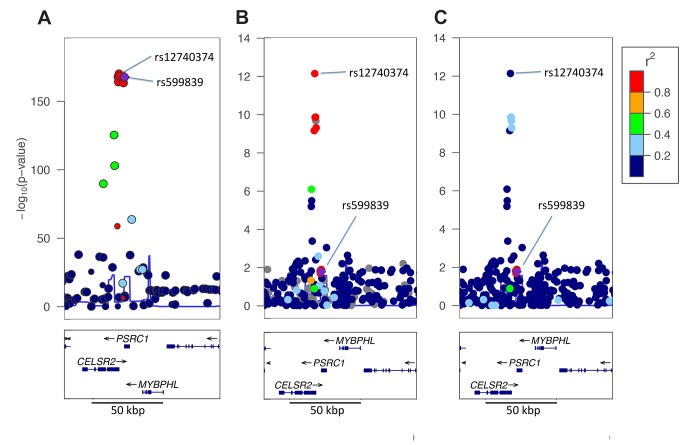
Dilution of effect size at PSRC1 for LDL. In panel (a), we show a locuszoom plot for the tagSNP rs599839 and LDL, using imputed data in a meta-analysis of more than 100,000 European individuals (image from the GLGC consortium locuszoom website [31]). The *y*-axis plots −log10(*p* value), which is a proxy for effect size, assuming similar allele frequencies. In panel (a) the size of the dot for each tagSNP represents the effective number of samples for which imputed data were available. The cluster of overlapping red dots at the top represents a bin of SNPs that are in very strong LD with the tagSNP, and have indistinguishable effect sizes in the EA study. Panel (b) shows data from our metabochip analysis in African Americans, but with dots color-coded using LD from the EA population. The scale of the *y*-axis has changed due to dramatically different sample sizes, but *p* value is still a useful proxy for effect size. Note how the tagSNP and several strongly associated SNPs (red data points) have effect sizes indistinguishable from background, while several other EA strongly associated SNPs remain significant, including rs12740374, the strongest signal in our data. Panel (c) shows our metabochip data again, but now color coding LD with the tagSNP rs599839 in our AA samples, rather than using EA LD. Rs599839 continues to tag several SNPs strongly in AA, and these are all among the SNPs with nonsignificant effect sizes in AA, while the SNPs with strongest residual signal are weakly tagged in AA. These data suggest that rs12740374 is the functional SNP; if so, then differential LD between rs12740374 and rs599839 in EA (r^2^>0.8) and AA (r^2^<0.2) would explain the diluted effect observed at rs599839 in AA.

In order to measure the contribution of differential LD to dilution, we need a model of how changes in LD between tagSNP and a functional variant would be expected to alter the observed effect size at the tagSNP, assuming that the effect size at the functional variant is the same in both populations. Given a functional SNP (fSNP) and an associated tagSNP, linkage disequilibrium between the two SNPs can be described as the measurement error introduced by genotyping the tagSNP, rather than genotyping the fSNP directly. As such, by appealing to prior work on regression dilution bias, it can be shown that the effect size *β′* at the tagSNP is related to the effect size *β* at the fSNP by the following equation: 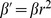 (see Text S1 for details). Thus, assuming that the effect size at the fSNP is constant between populations, when linkage disequilibrium between tagSNP and fSNP is weaker in a given population, we expect to see a greater degree of dilution bias for the estimated tagSNP effect size. Rearranging this equation, 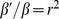. Extrapolating to compare the degree of dilution bias between AA and EA populations, we expect changes in linkage disequilibrium across populations to be reflected by changes in relative effect size:
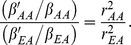
Assuming the effect size of the functional variant is the same in both populations, this reduces to:
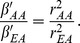



The above equation allows us to directly compare the observed distribution of relative effect sizes at the tagSNPs in AA and EA (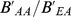) against the relative strength of tagging in AA and EA (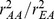). Considering the subset of index tagSNPs in regions that were present on the Metabochip, we observed 51 index tagSNPs that fell into 47 independent loci on the Metabochip. We identified the set of SNPs tagged by each index tagSNP at r^2^>0 .8 in an EA population [29],[30], yielding a total of 1,093 tagged SNPs for the 51 index tagSNPs. For each of these 1,144 SNPs, we then calculated 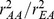. Let this represent the expected distribution of differential LD between AA and EA. Next, we calculated 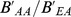 for the subset of 40 of the 51 index tagSNPs that replicated at q = 0.05 in EA, truncating at 0 if the signs were opposite between populations. These two distributions (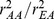 in all 1,144 SNPs versus 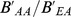 for the 40 index tagSNPs) were not significantly different by two-tailed *t* test. Thus, we cannot reject the hypothesis that the observed dilution bias in AA effect sizes at the index tagSNPs is consistent with the observed distribution of differential LD between the two populations. A single-locus example of the potential for differential LD to contribute to diluted effect sizes is shown in Figure 2.

Considering the 12 SNPs showing differential effect size in AA, regions spanning 11 were present on the Metabochip (Table S3). Before comparison with EA, we compared the observed effect sizes at the index tagSNPs in the full AA sample and the subsample of AAs genotyped on the Metabochip (AA_mchip_). Three of the index tagSNPs failed to genotype on the Metabochip, leaving eight index tagSNPs for this direct comparison (Table S4). No significant allele frequency differences were observed between the AA_mchip_ subset and the full AA population, consistent with AA_mchip_ being a representative subsample. A significantly inconsistent and diluted effect size in AA_mchip_ compared to EA was still observed for five of these eight tagSNPs (*p*<0.05, Table S4). The index tagSNPs without a significant difference likely reflect reduced power to detect the differential effect size in the AA_mchip_ subsample, as these three index tagSNPs also had the least significant differential effect when comparing the full PAGE AA subpopulation against EA.

**Figure 3:**
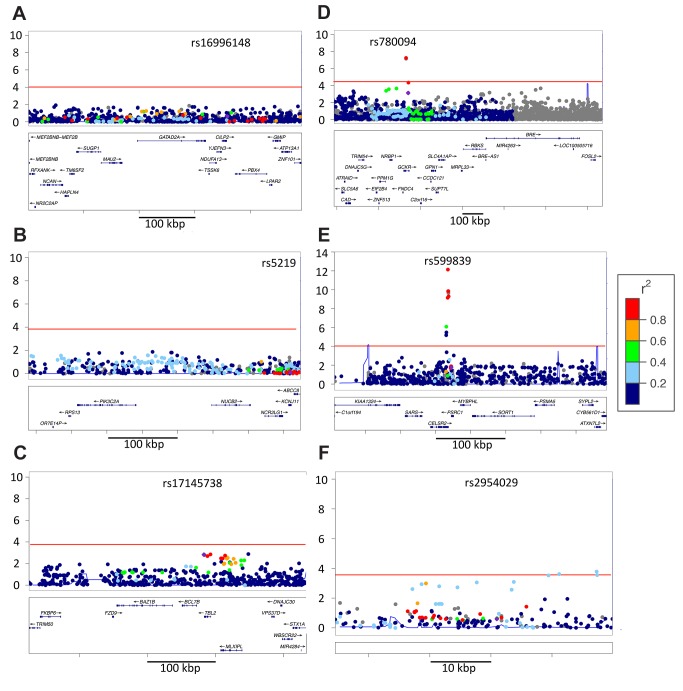
Examples of loci without evidence of association in AA_mchip_ or fine mapping EA signal. (a) At rs16996148 (CILP2/LDL) we are reasonably well powered, and no significant associations were observed in AA, suggesting that either the associated variant, or the synthetic allele that tags it is EA-restricted. Similar null results at (b) rs5219 (KCNJ11/T2D) and (c) rs17145738 (MLXIPL/logTG) were underpowered to draw strong conclusions. (d) At rs780094 (GCKR/logTG) and (e) rs599839 (PSRC1/LDL) the index tagSNP from EA showed significantly diluted signal in AA (purple dot). However, in each region a tagged SNP showed an effect size consistent with the EA index tagSNP, and after adjustment for this variant no residual evidence for association was observed at any additional variants in the region. (f) At rs2954029 (TRIB1/logTG) a similar effect was observed, save for the fact that the strongest AA association was imperfectly tagged in EA (r^2^ = 0.33).

**Figure 4:**
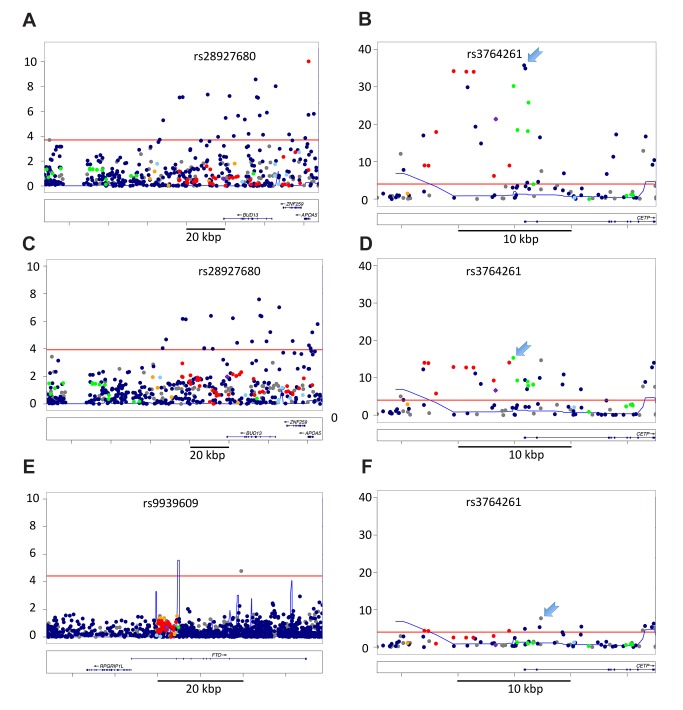
Examples of secondary alleles in the AA population. (a) At rs28927680 (APOA1/C3/A4/A5 gene cluster, logTG) the index tagSNP fine maps (red point in upper right of a). Panel (b) shows residual signal in the same region after adjustment for genotype at this variant, and significant secondary signals are observed. (c) At FTO, the SNPs tagged by rs9939069 in EA are all null in the subsample, but a secondary association is observed at very low frequency SNP (rs75569526, MAF 1% in AA_mchip_). In this example the secondary SNP is the only significant association in the region from our subsample analysis. Panels (d–f) illustrate multiple, independent associations at CETP. At CETP, the significant residual signal after adjusting for the best signal in each EA-tagged bin (Figure S2) is consistent multiple factors that might contribute to differential signal in the region. The number of independent statistical associations observed within the locus is a rough proxy for the number of functional alleles. Here we show a series of LocusZoom plots sequentially adjusting results for the SNP with the strongest observed association in the previous cycle. LD in EA samples is color coded relative to rs3764261 in all panels, and the region-wide threshold for significance after Bonferroni adjustment for the 84 SNPs genotyped in the 25 kb region (residual *p*<1.1 * 10^−4^) is shown as a horizontal red line. (d) CETP/HDL regional data adjusted only for ancestry. The strongest observed association at rs17231520 is indicated with an arrowhead. (e) After adjustment for genotype at rs17231520, the strongest residual association at rs4783961 is indicated with an arrowhead. (f) After adjustment for genotype at rs17231520 and rs4783961, the strongest residual association is still significant. These results suggest the presence of at least three statistically independent associations with HDL in the CETP region, in the AA population. Assuming that the functional variation has been directly genotyped, rather than tagged by LD, this would indicate the presence of at least three functional alleles, clustered within a 5 kb window spanning the putative CETP promoter region.

The Metabochip genotype data allowed us to evaluate regions spanning each of the 11 variants for the underlying contributions of population-specific alleles, differential tagging, and secondary alleles to differential effect sizes. Detailed discussion of each locus is provided in Text S1. In summary, the 11 SNPs fell in 10 Metabochip regions, so all SNPs in each of the 10 regions were assessed for association with the reported trait in AA_mchip_. The threshold level for significance within each region was conservatively adjusted for multiple testing by Bonferroni adjustment for the number of SNPs successfully genotyped on the Metabochip within the region, with minor allele frequency greater than 1% in the AA_mchip_ sample. For example, the Metabochip region spanning CETP contained 84 SNPs, so our significance threshold for that region was *p*<0.05/84 = 1.1*10^−4^. One locus (*APOE*) could not be dissected confidently as LD data for the index tagSNP were not available in EA, and two loci were underpowered to draw strong conclusions, as evidenced by the failure of any variant in the region to show a significantly inconsistent effect with the index tagSNP effect in EA. Among the remaining seven loci, we observed one clear example of a diluted signal consistent with EA-specific functional alleles, either common or synthetic (Figure 3a), and five loci showed patterns consistent with fine-mapping of the index tagSNP bin (Figure 3d–f, Figure 4a, 4d). One of these fine-mapped the EA association to a variant that was not strongly associated with the index tagSNP in EA (r^2^<0.5, Figure 3f), potentially consistent with a synthetic allele in EA. We also observed statistically significant secondary functional alleles at three loci (Figure 4).

Thus, although the overall pattern of effect dilution in AA is consistent with expectations on the basis of differential LD patterns between AA and EA populations, putative examples of EA-specific alleles and secondary alleles in AA were also observed. A contribution from synthetic alleles cannot be excluded, and may well account for the EA-specific allele at CILP2 (Figure 3a). However, at half of the 10 loci we observed at least one of the tagged SNPs in EA that showed an effect size in AA consistent with the effect size at the tagSNP in EA. These examples of fine-mapping EA signal suggest that at least half of EA GWAS signals tag a common, functional variant. The observed excess of dilution effects in AA (as compared to other non-EA populations) suggests that African-descended populations will be the most useful single subpopulation for fine-mapping of EA GWAS associations, although the significant trend toward excess dilution in HA and NA populations (Table S5) suggests that trans-ethnic fine-mapping may prove more powerful than fine-mapping with any single non-EA population.

In conclusion, we have assessed the generalization of GWAS associations from EA populations across five clinically relevant traits, in five non-EA populations. Our results demonstrate that although most EA GWAS findings can be expected to show an effect in the same direction for non-EA populations, a significant fraction of GWAS-identified variants from EA will exhibit differential effect sizes in at least one non-EA population, and these differential results will be far more frequent in the AA population. These findings suggest that expanded GWAS and fine-mapping efforts focused on non-EA populations, especially AA, will substantially enhance our understanding of the genetic architecture of common traits within non-EA populations. It will be particularly important to extend GWAS discovery efforts to non-EA populations if genetic risk prediction models using tagSNP genotypes demonstrate clinical utility, because risk estimates derived from European GWAS clearly generalize imperfectly to non-EA populations. Our analyses suggest that variable LD in its many guises accounts for much of the heterogeneity of effect size at index tagSNPs, rather than any “true” differences in effect size between populations for the functional variants that were tagged. Thus, risk models derived directly from genotypes at functional variants (rather than tagSNPs) may generalize more effectively to non-EA populations.

## Materials and Methods

### Selecting Index SNPs from Prior Reports

Traits considered were those for which more than 10 GWAS-identified variants were genotyped in the first year of PAGE. Variants considered for this analysis included 13 previously reported to associate with body mass index, 20 for type 2 diabetes, 27 for HDL, 19 for LDL, and 14 for triglycerides. Eleven of these GWAS-identified variants were previously reported to associate with more than one trait in EA (Table S1), so we constrained the analysis of each such SNP to whichever trait had the most significant association (smallest *p* value) in the PAGE EA population, leaving a panel of 82 unique variants.

Because highly correlated SNPs might overweight specific results toward a specific trait or gene, we extracted a subset of minimally correlated index GWAS-identified variants from this panel of 82. At each step, we added the SNP with the most significant association in PAGE EA to a list of index SNPs, and then filtered the remaining SNPs not yet in the index list to exclude those exceeding r^2^ = 0.2 in the PAGE EA population with any index tagSNP. The panel of 82 SNPs was recursively filtered in this manner, leaving a final panel of 69 index SNPs, each of which was minimally associated with any other index SNP in the PAGE EA (r^2^<0.2). One additional SNP (rs11084753) was removed from the analysis due to concerns regarding power to replicate, leaving a final panel of 68 index SNPs for analysis, including seven index SNPs for BMI, 18 for HDL, 15 for LDL, nine for triglycerides, and 19 for T2D (Table S2).

### Assessing Power to Replicate Previous Reports

Power estimates are taken directly from Fesinmeyer et al. [13] for BMI, Dumitrescu et al. [15] for lipids, and Haiman et al. [14] for T2D. For details, see the original publications.

### Defining Generalization results for Each SNP

In order to assess the generalization of effects to each population, we used effect sizes (β) and standard errors derived from minimally adjusted (age, sex, and study), ancestry-specific meta-analyses described in the primary PAGE publications [15],[13],[14]. Using these data, we tested two hypotheses: first, that the GWAS-identified variant has no effect in the non-EA population (i.e., the coefficient **ß_pop_** = 0 in a linear or logistic regression model), and second, that the effect size in the non-EA population is the same as the effect size in EA (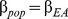). The first hypothesis was tested by assuming the estimate 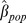 is normally distributed (which is reasonable as sample sizes exceeded 500 for all populations) and calculating the probability that 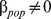 given 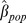 and the standard error of 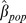. The second hypothesis was tested by defining 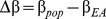 and calculating the probability that 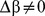, again assuming 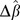 to be normally distributed. These tests are all carried out at a nominal significance level of 0.05, as we see them as the (single) test that an investigator may carry out to validate a result first observed in EA in another ethnic group, and then significance was assigned using the Benjamini-Hochberg method at a false discovery rate of 5%.

A reasonable concern in these analyses is that population stratification can distort effect size estimates in some circumstances. Some of the effect sizes from trait-specific PAGE manuscripts were not adjusted for genetic ancestry, due to either availability of data [15] or informed consent in specific populations [13],[14]; where available and allowed we have used the ancestry adjusted effect sizes. Both ancestry adjusted and unadjusted data were available for the PAGE obesity analysis [13], where ancestry adjustment did not significantly alter effect size estimates.

## Supporting Information

Figure S1 **Generalization analysis in the PAGE populations.** We plot the ratio of ß_pop_/ß_EA_ on the *y*-axis as an indicator of both consistency of direction (positive values are consistent with effects in the same direction) and relative magnitude of effect (consistent but weaker effects in the non-EA will have ratios between 0 and 1). We plot the coded allele frequency (CAF) in the non-EA population on the *x*-axis, as a proxy for power to replicate in the available sample size. Data points are colored as follows: ambiguous SNPs are light blue (ß_pop_ = 0 and ß_pop_ = ß_EA_), strictly generalized SNPs are dark blue (ß_pop_≠0 and ß_pop_ = ß_EA_), differentially generalized SNPs are dark red (ß_pop_≠0 and ß_pop_≠ß_EA_), and differential SNPs are pink (ß_pop_ = 0 and ß_pop_≠ß_EA_). The *y*-axis has been constrained to (−4.4) for illustrative purposes; some loci yielded ß_pop_/ß_EA_ ratios outside this range, but p_EA_>0.05 for all of these. As expected, larger non-EA populations show less scatter in ß_pop_/ß_EA_ than the smaller non-EA populations (particularly Pacific Islanders), consistent with more precise estimates of ß_pop_ in the larger non-EA populations. A clear trend is observed toward ß_pop_/ß_EA_ ratios greater than zero in all populations, reflecting consistency of direction between EA and non-EA populations. An additional trend toward ratios greater than zero but less than one is observed in African Americans, representing the trend toward dilution in this population, relative to EA. No such trend is apparent in the other non-EA populations. Differential and ambiguous SNPs are observed throughout the CAF range, consistent with the assertion that these categories do not reflect a systematic bias toward underpowered, low-frequency variants.
(TIF)

Figure S2 **Multiple associations at CETP.** Two index tagSNPs with differential effect size were observed at CETP: rs3764261 and rs9989419. (a) CETP regional LocusZoom plot with LD in EA samples color coded relative to rs3764261. Although this index tagSNP exhibited differential effect (purple point indicated with arrow), several of the tagged SNPs (red data points near −log10(p) = 34, rs247616, rs247617, rs183130) exhibited effect sizes consistent with fine-mapping of this association (r^2^ = 0.99 in EA, r^2 = ^0.74 in AA). Interestingly, the strongest effect observed in the region was at a SNP uncorrelated with rs3764261 (rs17231520, r^2^_EA_<0.001). (b) Same plot, but adjusting genotype at rs274616 (the best signal from a tagged SNP in the EA rs3764261 bin). The signal from tagged SNPs has clearly been reduced to background levels, and residual signal is clearly visible for untagged SNPs. (c) Same plot, but now adjusting for genotype at rs274616 and rs193695 (the best signal from a tagged SNP in the EA rs9989419 bin). Again, significant residual signal is observed. Figures S2d–f show the same data, but with LD in EA samples color coded relative to rs9989419. Although rs9989419 failed to genotype on the Metabochip, a strongly tagged SNP is visible in (d). Although this variant was weakly tagged by rs3764261 (compare panel a with d), the association signal does not appear to be independent of rs3764261, as residual association is not significant at this variant after adjustment for rs247616 (e). Thus, there is clearly residual association at this locus after adjusting for both of the strongest EAtaggedSNPs, consistent with either additional functional variation in the region, differential tagging, or differential synthetic alleles at this locus.
(TIF)

Table S1 **Categorization of tagSNPs.** TagSNPs are categorized on the basis of the primary phenotype in the original GWAS report, and by whether these SNPs were categorized as index SNPs or not in the present analysis.
(XLSX)

Table S2 **Raw meta-analysis data for each of the index SNPs is given in each PAGE subpopulation, including the number of individuals in the subpopulation, the observed effect size and s.e., the** ***p*** **value associated with testing the hypothesis that the observed effect was significant within the subpopulation (p_Beta_<subpopulation>_ = _0), and the** ***p*** **value associated with testing the hypothesis of equal effect size in European and non-European populations (p_Beta_<subpopulation>_equal_Beta_Eur.Am.), as well as the generalization category for that SNP in the subpopulation (<subpopulation>_snpcat).**
(XLSX)

Table S3 **Details of the effect sizes and generalization results for the 12 tagSNPs with inconsistent effect size observed in the EA and AA PAGE populations, extracted from Table S2.**
(XLSX)

Table S4 **Summary of Metabochip variants that passed QC with minor allele frequency greater than 1% in the PAGE AAmchip subpopulation is given, along with the generalization results comparing only the index tagSNP from the AAmchip against PAGE EA.**
(XLSX)

Table S5 **Summary of dilution effects in subpopulations.** Data are shown first for the subset of 51 tagSNPs replicated in EA, then for all 68 tagSNPs in Table S2. Within each subpopulation, we first assessed the probability of the observed frequency of dilution (ß_pop_/ß_EA_<1) against the null hypothesis of no such trend, by chi-square test. Then we compared the frequency of dilution effects between other subpopulations and the AA subpopulation. As noted, we observed a significant excess of effects diluted toward the null (ß_pop_/ß_EA_<1) in AA, HA, and NA populations, and this trend toward diluted effects was significantly stronger in the AA subpopulation than in any other subpopulation.
(XLSX)

Text S1 **Supplemental methods.**
(DOCX)

## Abbreviations

AA: African Americans
APOA1/C3/A4/A5: Apolipoprotein AI, CIII, AIV, AV gene cluster
APOE/C1/C4: Apolipoprotein E, CI, CIV gene cluster
AS: Asian Americans
BMI: body mass index
CaLICo: causal variants across the life course
CELSR2: cadherin, EGF LAG seven-pass G-type receptor 2
CETP: cholesteryl ester transfer protein, plasma
CILP2: cartilage intermediate layer protein 2
EA: European ancestry
EAGLE: Epidemiologic Architecture for Genes Linked to Environment
FDR: false discovery rate
FTO: fat mass and obesity associated
GCKR: glucokinase (hexokinase 4) regulator
GWAS: genome-wide association study
HA: Hispanic Americans
HDL: high density lipoprotein
KCNJ11: potassium inwardly-rectifying channel, subfamily J, member 11
LD: linkage disequilibrium
LDL: low density lipoprotein
LIPC: lipase, hepatic
MEC: multi-ethnic cohort
MLXIPL: MLX interacting protein-like
NA: Native Americans
PAGE: Population Architecture using Genomics and Epidemiology
PBX4: pre-B-cell leukemia homeobox 4
PI: Pacific Islanders
PSRC1: proline/serine-rich coiled-coil 1
SNP: simple nucleotide polymorphism
T2D: Type 2 Diabetes
TG: triglycerides
TRIB1: tribbles homolog 1
WHI: Women's Health Initiative
