# Supplementary figures and images for: Generalization and Dilution of Association Results from European GWAS in Populations of Non-European Ancestry: The PAGE Study
Source: PLoS Biol. 2013 Sep 17;11(9):e1001661. doi: 10.1371/journal.pbio.1001661 (PMC3775722; doi:10.1371/journal.pbio.1001661)

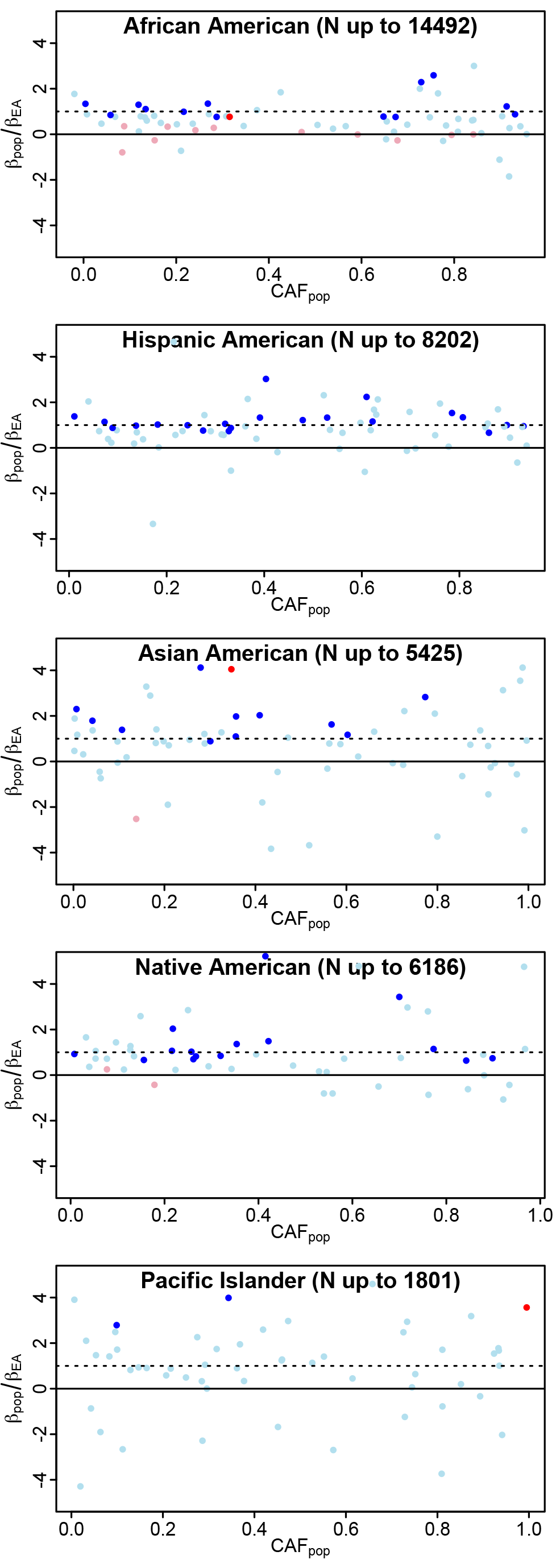

Supplement: Figure S1 — Generalization analysis in the PAGE populations. We plot the ratio of ßpop/ßEA on the y-axis as an indicator of both consistency of direction (positive values are consistent with effects in the same direction) and relative magnitude of effect (consistent but weaker effects in the non-EA will have ratios between 0 and 1). We plot the coded allele frequency (CAF) in the non-EA population on the x-axis, as a proxy for power to replicate in the available sample size. Data points are colored as follows: ambiguous SNPs are light blue (ßpop = 0 and ßpop = ßEA), strictly generalized SNPs are dark blue (ßpop≠0 and ßpop = ßEA), differentially generalized SNPs are dark red (ßpop≠0 and ßpop≠ßEA), and differential SNPs are pink (ßpop = 0 and ßpop≠ßEA). The y-axis has been constrained to (−4.4) for illustrative purposes; some loci yielded ßpop/ßEA ratios outside this range, but pEA>0.05 for all of these. As expected, larger non-EA populations show less scatter in ßpop/ßEA than the smaller non-EA populations (particularly Pacific Islanders), consistent with more precise estimates of ßpop in the larger non-EA populations. A clear trend is observed toward ßpop/ßEA ratios greater than zero in all populations, reflecting consistency of direction between EA and non-EA populations. An additional trend toward ratios greater than zero but less than one is observed in African Americans, representing the trend toward dilution in this population, relative to EA. No such trend is apparent in the other non-EA populations. Differential and ambiguous SNPs are observed throughout the CAF range, consistent with the assertion that these categories do not reflect a systematic bias toward underpowered, low-frequency variants. (TIF) [file pbio.1001661.s001.tif]

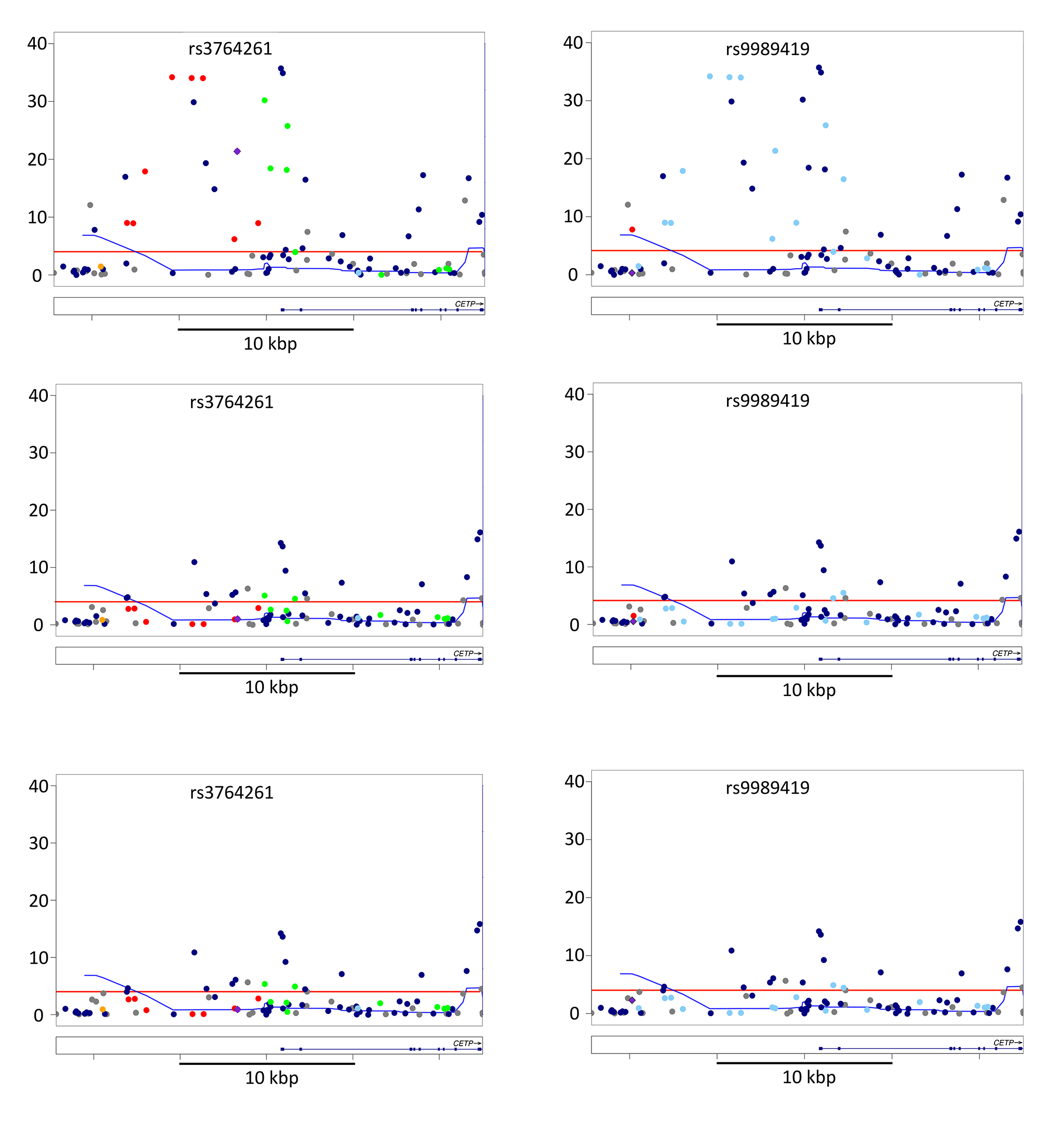

Supplement: Figure S2 — Multiple associations at CETP. Two index tagSNPs with differential effect size were observed at CETP: rs3764261 and rs9989419. (a) CETP regional LocusZoom plot with LD in EA samples color coded relative to rs3764261. Although this index tagSNP exhibited differential effect (purple point indicated with arrow), several of the tagged SNPs (red data points near −log10(p) = 34, rs247616, rs247617, rs183130) exhibited effect sizes consistent with fine-mapping of this association (r2 = 0.99 in EA, r2 = 0.74 in AA). Interestingly, the strongest effect observed in the region was at a SNP uncorrelated with rs3764261 (rs17231520, r2 EA<0.001). (b) Same plot, but adjusting genotype at rs274616 (the best signal from a tagged SNP in the EA rs3764261 bin). The signal from tagged SNPs has clearly been reduced to background levels, and residual signal is clearly visible for untagged SNPs. (c) Same plot, but now adjusting for genotype at rs274616 and rs193695 (the best signal from a tagged SNP in the EA rs9989419 bin). Again, significant residual signal is observed. Figures S2d–f show the same data, but with LD in EA samples color coded relative to rs9989419. Although rs9989419 failed to genotype on the Metabochip, a strongly tagged SNP is visible in (d). Although this variant was weakly tagged by rs3764261 (compare panel a with d), the association signal does not appear to be independent of rs3764261, as residual association is not significant at this variant after adjustment for rs247616 (e). Thus, there is clearly residual association at this locus after adjusting for both of the strongest EAtaggedSNPs, consistent with either additional functional variation in the region, differential tagging, or differential synthetic alleles at this locus. (TIF) [file pbio.1001661.s002.tif]
