# Supplementary material for: Generalization and Dilution of Association Results from European GWAS in Populations of Non-European Ancestry: The PAGE Study
Source: PLoS Biol. 2013 Sep 17;11(9):e1001661. doi: 10.1371/journal.pbio.1001661 (PMC3775722; doi:10.1371/journal.pbio.1001661)
Supplement: Text S1 — Supplemental methods. (DOCX) [file pbio.1001661.s008.docx]

Supplemental Methods

Effect sizes for lipids[[1](#_ENREF_1)], BMI[[2](#_ENREF_2)] and T2D[[3](#_ENREF_3)] were estimated in models that included estimated genetic ancestry, where available and when allowed by the informed consent protocols.

In order to formally assess the contribution of changes in LD between populations to observed differential effects, we draw from the statistical literature regarding regression dilution bias, reviewed in [[4](#_ENREF_4)]. In the generic sense, dilution bias drives the estimated regression coefficient toward 0 when the independent variable is measured with error. Applied specifically to tagSNPs, linkage disequilibrium between tagSNP and fSNP is a measure of the error introduced by assessing effect size at the tagSNP, rather than directly at the fSNP.

Adapting notation and text from Frost and Thompson [[4](#_ENREF_4)], assume $y_{i}=\alpha+\beta x_{i}+\varepsilon$, where $x_{i}\sim N(\mu,\sigma_{b}^{2})$ represents the independent variable measured without error, and $\varepsilon\sim N(0,\phi^{2})$. Now assume that the independent variable is measured with error (*w_i_*), such that $w_{i}=x_{i}+u_{i}$ , with $u_{i}\sim N\left( 0,\sigma_{w}^{2} \right)$representing random error. "If *u_i_*, $\varepsilon$, and *x_i_* are independently distributed it is known (Snedecor and Cochran 1967) [...] that the regression of *y_i_* on *w_i_* is linear as

$y_{i}=\alpha^{'}+\beta^{'w_{i}}+\gamma_{i}$ , with $\gamma_{i}\sim N(0,\varphi^{2})$, where $\beta^{'}=\beta\sigma_{b}^{2}/(\sigma_{b}^{2}+\sigma_{w}^{2})$. "

Applying the above to the case of fSNPs and tagSNPs, let *x_i_* represent genotype at the fSNP and *w_i_* be genotype at the tagSNP. Although allele counts are not normally distributed, a similar reduction in the estimated regression coefficient is expected. Linkage disequilibrium measured as r^2^ between fSNP and tagSNP can be described as the proportion of the variance in genotype *x_i_* explained by genotype *w_i_*, and is therefore a direct estimate of $\sigma_{b}^{2}/(\sigma_{b}^{2}+\sigma_{w}^{2})$. Thus, for a tagSNP and fSNP with known *r^2^*, $\beta^{'}=\beta r^{2}$.

Supplemental Discussion

Using the region-specific thresholds described in the main text, we observed significant associations with one or more SNPs in seven of the ten regions, with no significant associations observed in three regions (**Figure 2a, 2b** and **2c** respectively). Two of these regions appear to be underpowered to dissect the differential effect size, as the hypothesis of a consistent effect size cannot be rejected in the AA_mchip_ subsample, but at rs16996148 not even the strongest AA_mchip_ association with LDL in the region was consistent with the observed EA effect size (**2a**). This suggests that the functional variation tagged by rs16996148 in EA may not be present in the AA population at more than 1% frequency.

Considering the remaining seven regions, LD calculated in the EA population between the index tagSNP and all other variants in the region allowed us to assess whether any tagged variant in EA (r^2^ > 0.2, an "EAtaggedSNP") showed an effect size in AA_mchip_ consistent with the index tagSNP effect size in EA. Three loci an EAtaggedSNP was observed with an effect consistent with the index tagSNP, and no residual significant associations were observed after adjustment for genotype at the EAtaggedSNP with the strongest association (**Figure 2d-f**). These observations are consistent with fine-mapping of the original EA association within a bin of taggedSNPs, indicating that differential LD was the major source of the observed differential effect size. Fine-mapping also contributed to, but did not entirely account for, inconsistent effects at several additional loci (**Figure 3a, 3d-f**).

We observed evidence of independent, secondary alleles associated with the same phenotype in at least three of the loci (**Figure 3**). At rs28927680 (the ApoA1/C3/A4/A5 gene cluster associated with logTG, **Figure** **3a**) we observe evidence for fine mapping of the index association, but in models adjusting for genotype at the MetabochipSNP with the strongest association in the region, we observe additional MetabochipSNPs with significant residual association and no significant LD with the index tagSNP (**Figure 3b**), indicating that at least one additional, independent signal is present in the AA population. At rs9969039 (FTO/BMI, **Figure 3c**), the SNPs tagged by rs9939069 in EA are all null, but an association is observed for a very low frequency SNP in AA (rs75569526, MAF 1% in AA_mchip_). The AA SNP is therefore unlikely to explain the EA association, and is the only significant association in the region.

Rs4420638 failed to genotype on the Metabochip, and the known functional variant associated with LDL, rs429358 which defines APOE4, also failed. In the absence of LD data for these SNPs, LocusZoom plots are not shown for this region. Given that key functional variants failed to genotype, it was not possible to confidently dissect the cause of differential effect sizes at this locus. However, the well-known rs7412 SNP that defines the APOE2 isoform yielded far the strongest association in this region, and after adjusting for this variant, significant evidence persisted, consistent with additional effect from at least one additional variant (likely the APOE4 variant).

Finally, rs9989419 and rs3764261 are both located within the Metabochip region encompassing CETP that associates with HDL. These two variants showed independent evidence for association with HDL in both the EA and AA PAGE populations, but with significantly weaker effects in the AA population. Locuszoom for the region shows a large number of very significant associations, many but not all of which are tagged by one of the two index tagSNPs (**Supplemental** **Figure 2a/2d**). Both signals showed significant evidence for fine mapping, so the results were adjusted for genotype at rs274616, the strongest EAtaggedSNP in the EA rs3764261 bin (**Supplemental Figure 2b/2e**). Significant residual association was observed, including at variants tagged by rs9989419, so the results were further adjusted for genotype at rs193695, the strongest remaining EAtaggedSNP in the EA rs9989419bin (**Supplemental Figure 2c/2f**).

Clearly, significant residual evidence was observed for association after adjusting for the two index tagSNP bins at CETP. The strongest observed association in AA was tagged by neither index tagSNP, but such findings could be consistent with differential tagging of functional alleles by synthetic alleles, additional functional alleles, or a combination of both. The number of independent signals in the region cannot fully resolve the potential contribution of synthetic alleles, but can be used to assess whether more than two independent signals truly exist. The CETP data were iteratively reanalyzed, at each step adding the genotype at the SNP with the strongest remaining significant association to the model, in order to estimate the number of independent association signals. Starting from signal unadjusted for any genotype (**Figure 3d**), in the first cycle the strongest association in the region was added to the model (rs17235120, **Figure 3e**), and in the second cycle the strongest residual signal was added to the model (rs4783961, **Figure 3f**). This stepwise analysis suggested more than three independent signals in the region, so additional functional alleles may be contributing to the differential effects observed at this locus.

1. Dumitrescu, L., et al., *Genetic determinants of lipid traits in diverse populations from the population architecture using genomics and epidemiology (PAGE) study.* PLoS Genet, 2011. **7**(6): p. e1002138.

2. Fesinmeyer, M.D., et al., *Genetic Risk Factors for BMI and Obesity in an Ethnically Diverse Population: Results From the Population Architecture Using Genomics and Epidemiology (PAGE) Study.* Obesity (Silver Spring), 2012.

3. Haiman, C.A., et al., *Consistent Directions of Effect for Established Type 2 Diabetes Risk Variants Across Populations: The Population Architecture using Genomics and Epidemiology (PAGE) Consortium.* Diabetes, 2012. **61**(6): p. 1642-7.

4. Frost, C. and S.G. Thompson, *Correcting for regression dilution bias: comparison of methods for a single predictor variable.* Journal of the Royal Statistical Society: Series A (Statistics in Society), 2000. **163**(2): p. 173-189.
